# Decreased Global Network Efficiency in Young Male Smoker: An EEG Study during the Resting State

**DOI:** 10.3389/fpsyg.2017.01605

**Published:** 2017-09-15

**Authors:** Shaoping Su, Dahua Yu, Jiadong Cheng, Yajing Chen, Xiaohua Zhang, Yanyan Guan, Yangding Li, Yanzhi Bi, Ting Xue, Xiaoqi Lu, Kai Yuan

**Affiliations:** ^1^Inner Mongolia Key Laboratory of Pattern Recognition and Intelligent Image Processing, School of Information Engineering, Inner Mongolia University of Science and Technology Baotou, China; ^2^School of Life Science and Technology, Xidian University Xi'an, China; ^3^Engineering Research Center of Molecular and Neuro Imaging, Ministry of Education Xi'an, China; ^4^Guangxi Key Laboratory of Multi-source Information Mining and Security, Guangxi Normal University Guilin, China

**Keywords:** smoking, electroencephalogram (EEG), resting state, minimum spanning tree (MST), leaf fraction

## Abstract

Previous electroencephalogram (EEG) studies revealed reduced spectral power during the resting state in smokers. However, few studies investigated the changes of global brain networks during the resting state in young smokers by EEG. In the present study, we used minimum spanning tree (MST) to assess the differences of global network efficiency between young smoker (*n* = 20) and nonsmokers (*n* = 20). Compared with healthy nonsmokers, young smokers showed decreased leaf fraction, kappa value, increased diameter and eccentricity value in alpha band (*r* = 0.574, *p* = 0.008), which suggested the global network efficiency was decreased in young smokers. We also found positive correlation between leaf fraction and onset time of smoking in smokers. These results provided more scientific evidence of the abnormal neural oscillations of young smokers and improved our understanding of smoking addiction.

## Introduction

Annual smoking-attributable deaths was about 6 million in the world including one million in China (World Health Organization, 2013). “China Report on the Health Hazards of Smoking” in 2012 indicated China had more than 350 million smokers, of which 14 millions were young smokers. The prevalence of smoking during adolescence was higher than other age stages. People during the period from adolescence to young adulthood are undergoing a series of continuous and important physiological development. Therefore, young people are relative sensitive to outside influences, especially the effects of nicotine exposure (Gulley and Juraska, 2013; Lydon et al., 2014).

During the resting state, the brain may be conceived as a complex functional network constituted by correlated spontaneous fluctuations occur within brain regions (Seeley et al., 2009). Electroencephalography (EEG) had been employed to measure ongoing oscillation of neural electrical activity in the absence of an overt task or stimulation (Delbeuck et al., 2003; Stam et al., 2007). By using spectral analysis of neural oscillations in EEG, previous resting state studies had found decreased theta and delta power, increased beta power and inconsistent results in alpha band.in smokers (Rass et al., 2015). Graph theoretical network analyses (GTA) of EEG signal during the resting state was used to investigate the integrity and efficiency of global brain networks in brain diseases, by assessing the topology characteristics of global brain networks (Delbeuck et al., 2003; Lee et al., 2006; Leistedt et al., 2009; Fraga González et al., 2016). The findings improved our understanding of neural mechanisms of brain diseases from whole brain network. However, few study used EEG to investigate the changes of global brain networks during the resting state in young smokers.

Spectral analysis provided us a way to detect the changes of spectral power in different neural oscillations, which were related with the cortical arousal or activation (Rass et al., 2015). Compared with traditional EEG methods, the phase lag index (PLI) assessed the consistency of phase lags between time series, which may avoid the effect of spurious interactions caused by common reference effects or volume conduction (Stam et al., 2007; Yuan et al., 2016). Moreover, the Minimum Spanning Tree (MST) analysis can be used to derive the connectivity matrix of functional network topology, which contains the strongest connections to avoid the problem of arbitrary thresholding of connectivity values for network reconstruction and preserve important information about network organization (Boersma et al., 2013; Tewarie et al., 2015). Therefore, in the current study, we constructed global network of EEG in different spectrum bands to investigate the changes of functional network topology during the resting state in young smokers. We used PLI to construct the matrix of functional connectivity and employed MST to reconstruct whole-brain functional connectivity networks of young smokers in different frequency bands (Brody et al., 2007; van Dellen et al., 2014, 2015; Tewarie et al., 2015; Fraga González et al., 2016). Main four EEG bands (delta, theta, alpha, and beta) with global brain recording sites were analyzed to examine the differences of global brain network topology between young smokers and nonsmokers. It is hoped that our study could improve the understanding of neural mechanisms in smoking.

## Materials and methods

### Ethics statement

The current study was consistent with the Declaration of Helsinki and was approved by the Medical Ethical Committee of the First Affiliated Hospital of Baotou Medical College, Inner Mongolia University of Science and Technology. All subjects wrote informed consent when they had understood the purpose of our study.

### Participants

All participants were recruited from Inner Mongolia University of Science and Technology by advertisement. The exclusion criteria were any neurological illness, psychopathological diseases or epilepsy, drug abuse/dependence (excluding nicotine) based on DSM-V criteria and any current or past medications that may affect cognitive function, claustrophobia or not right-handed. Moreover, smokers were enrolled with regular smoked for more than 2 years and met the requirements of the DSM-IV criteria to nicotine dependence. The nicotine dependence was assessed by the Fagerström Test for Nicotine Dependence (FTND) (Fagerstrom and Schneider, 1989). Nonsmokers smoked less than 3 cigarettes in life. At last, twenty young male smokers (age: 20.95 ± 1.2 years) and 20 matched nonsmokers (age: 20.29 ± 1.1years) participated in our study. The smokers were asked to refrain from smoking about 60 min between the last cigarette and the acquisition of EEG data (average duration of abstinence before acquisition: 50.1 ± 5.8 min) (Yuan et al., 2016). The demographic characteristics of participants were presented in Table 1.

**Table 1:** Demographic characteristics of young smokers and nonsmokers.

	**Control (*N* = 20)**	**Smoker (*N* = 20)**	**Analysis**	***P***
Age	20.29 ± 1.1	20.95 ± 1.2	*F*_(1, 39)_ = 1.951	1.70
Education (years)	14.38 ± 0.9	14.10 ± 0.8	*F*_(1, 39)_ = 2.348	0.13
Smoking duration (years)		4.24 ± 1.8		
Cigarettes per day		14.05 ± 3.9		
Pack-years		3.00 ± 1.8		
Onset time (age)		15.30 ± 1.8		
FTND total score		4.60 ± 1.5		
CO ppm	2.52 ± 0.9	8.20 ± 2.8	*F*_(1, 39)_ = 16.91	*P* < 0.01

*Values are expressed as means ± standard deviations*.

*Pack-years: smoking years × Daily consumption/20*.

*FTND: Fagerström Test for Nicotine Dependence*.

### EEG recording and signal processing

Prior to EEG recording, the expiratory carbon monoxide (CO) levels of participants were measured by using the Smokerlyzer system (Bedfont Scientific Ltd., Rochester, UK). The EEG data acquisitions were recorded using digital BrainAmp MR plus amplifiers (Brain Products GmbH. Munich. Germany) with Ag/AgCl electrodes at the 64 scalp sites according to the International 10/20 System. The horizontal electro-oculogram (HEOG) and vertical electro-oculogram (VEOG) recording were using two active Ag/AgCl electrodes placed at outer canthus of right eye and above the left eye. Electrical signal were digitized with a sample rate of 512 Hz and all electrodes impedance was kept below 5KΩ. Offline data was analyzed by Brain Vision Analyzer 2 (Brain Products Inc. Munich, Germany). The reference electrodes were the bilateral mastoids (TP9 and TP10). EEG data were filtered by using 0.5–70 Hz (IIR filter 24 dB/double frequency) band pass filter. In addition, independent component analysis (ICA) is used for the automatic correction function in Brain Visual Analyzer 2. The 2 min EEG data was segmented into 8s epochs (4,096 sample points per epoch) which were visually inspected for eye blinks or other artifacts. For each subject, 10 artifact-free epochs were chosen and exported to ASCII files.

After an initial stage of processing for 2 min EEG data, frequency bands were filtered as delta band (0.5 ~ 4 Hz), theta band (4 ~ 8 Hz), alpha band (8 ~ 13 Hz) and beta band (13 ~ 25Hz). The EEG data was entered into Brainwave soft (Brainwave 0.9.151.7.2 developed by C.S.; freely available at http://home.Kpn.nl/stam7883/brainwave.html).

### Functional connectivity

The functional connectivities of 64-channels EEG time series were analyzed by the phase lag index (PLI), a method of the asymmetry of the distribution of phase differences (Δϕ(t_k_)=1,2,3,…,N) between two time-series (Stam et al., 2007). The PLI measures phase synchronization based on the asymmetry of distribution of instantaneous phase differences between two signals, which is determined by using the analytical signal based on the Hilbert transformation (Stam et al., 2007). Synchronization between two time series is calculated by the consistency of the phase lag of the non-zero to another signal (van Dellen et al., 2009). The instantaneous phase difference of each sampling point is analyzed by using the concept of signal analysis and Hilbert transform. The PLI have is high reliable of to measure asymmetric distribution measure functional interaction of these signal, which may be unaffected cannot make clear by volume conduction about a nonzero phase lag (van Dellen et al., 2014).

The PLI collected time series between two signals is used formula follow:

$$\begin{array}{l} \text{PLI}=|\langle \text{sign}\left[\text{sin}\left(\Delta \varphi \left(t_{k}\right)\right)\right]\rangle | \end{array}$$

In here, || means absolute value, 〈〉 is use mean value and the PLI value range of 0 (no phase locking) to 1(complete synchronization) (Pischon et al., 2010).

### Minimum spanning tree

**Figure 1:**
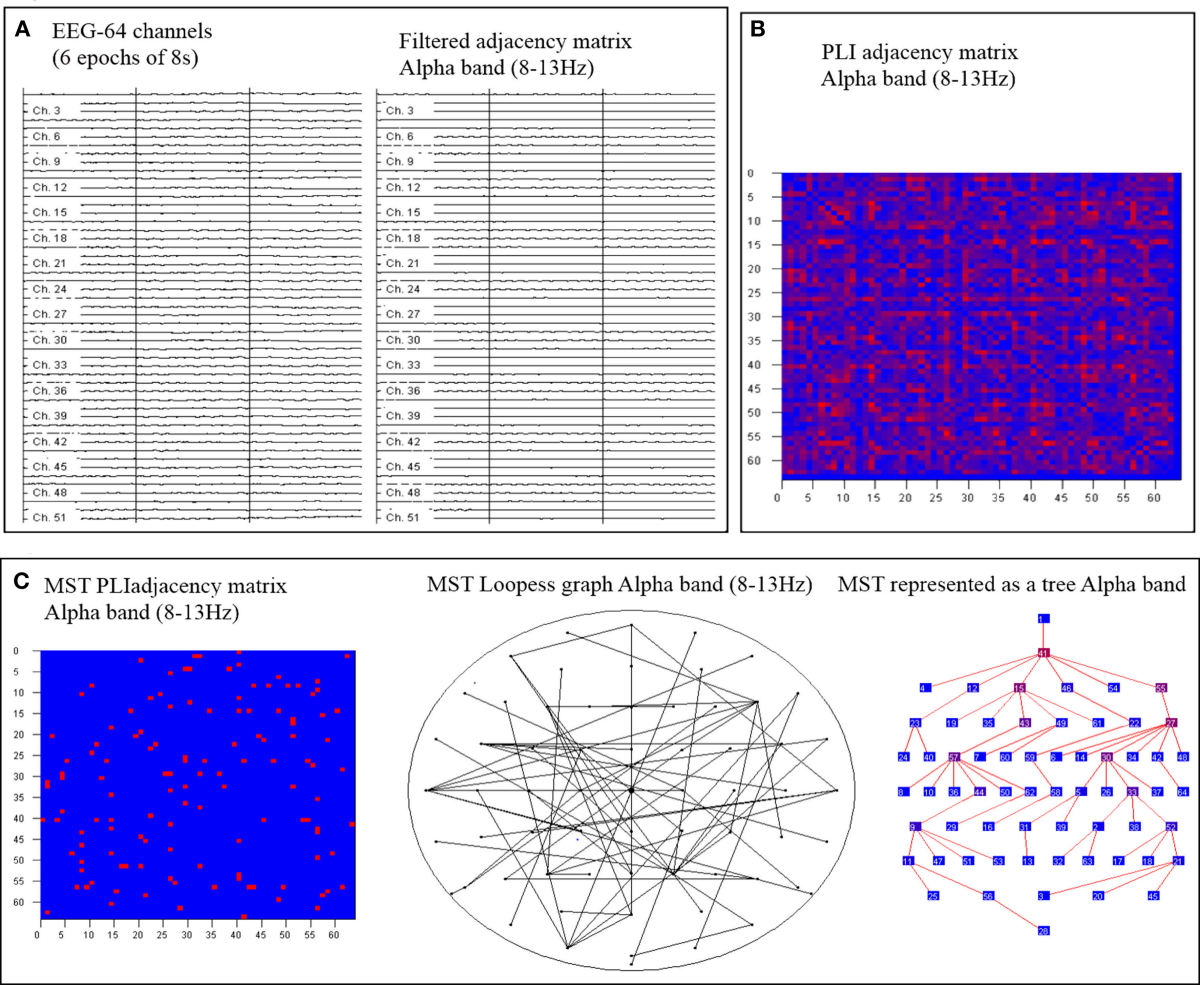
An overview of the steps. First, artifact-free epochs are filtered for each frequency band **(A)**. Secondly, the functional connectivity matrix based on Phase lag index (PLI) is calculated for each frequency band and epoch **(B)**. Finally, using MST construct global networks and the tree view shows the hierarchical structure of the graph **(C)**.

The 64 × 64 weight adjacency matrix was obtained by PLI method. Then, MST method was used to analyze the weight matrix. The MST is not form a loop which is connected min edge *M* = N−1 and weight edges in the tree. In addition, using MST come into being a tree is will unique if weight graph is unique (Mare, 2008). To construct brain network defined node and edge is importance, so we defined 64 channels as nodes. The edges were detected by using MST method connection. The whole experiment processes were described in Figure 1.

The following global metrics were calculated:

- Degree (s): the tree node degree.
- Leaf fraction: number of tree nodes with exactly one connection to any other node.
- Diameter (d): the largest distance between any two nodes.
- Eccentricity: the shortest path distance between node i and any other tree node.
- Betweenness centrality (BC): a network hub metric (Betzel et al., 2011) by $\text{b}{\text{c}}_{i}=\frac{1}{\left(n-1\right)\left(n-2\right)}\overset{n}{\underset{j\neq k,k\neq i,j\neq i}{\sum }}\frac{g_{jk}\left(i\right)}{g_{jk}}$ where g_jk_ is the shortest path between two tree nodes and g_*jk*_(*i*) is the number of those nodes that pass through node i.
- Hierarchy (T_H_): captures the ratio between a small diameter on one hand and overloading of hub nodes (Stam, 2014) by 2: ${\text{T}}_{\text{H}}=\frac{{\text{N}}_{leaf}}{2\cdot M\cdot BC_{\max}}$.
- kappa (κ): captures the broadness of the degree distribution: $k=\frac{\langle s\cdot s\rangle }{\langle s\rangle }$ (Stam and van Straaten, 2012; Stam et al., 2014).

### Statistical analysis

Two independent samples *t*-tests were used to examine the difference in the mean of two unrelated samples on the same variable by using SPSS 20.0 software. Pearson's correlation coefficients were calculated for performance of the Minimum spanning tree and smoking behaviors.

## Results

### Minimum spanning tree network analysis

**Figure 2:**
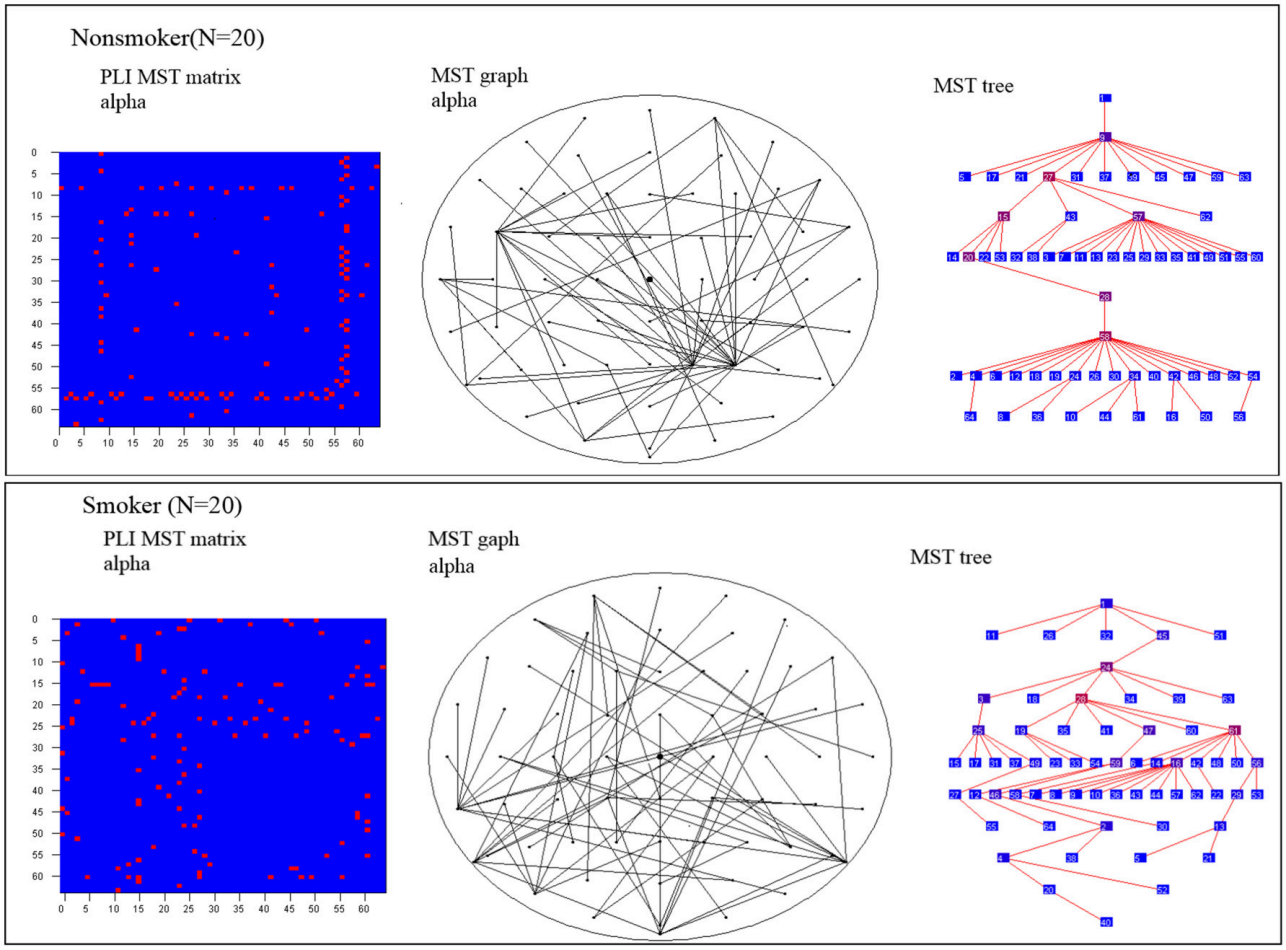
MST matrices and MST graph in scalp view and tree view for the alpha band for nonsmoker and smoker. For illustrative the MST method was difference brain networks in smoker compare with nonsmoker.

**Figure 3:**
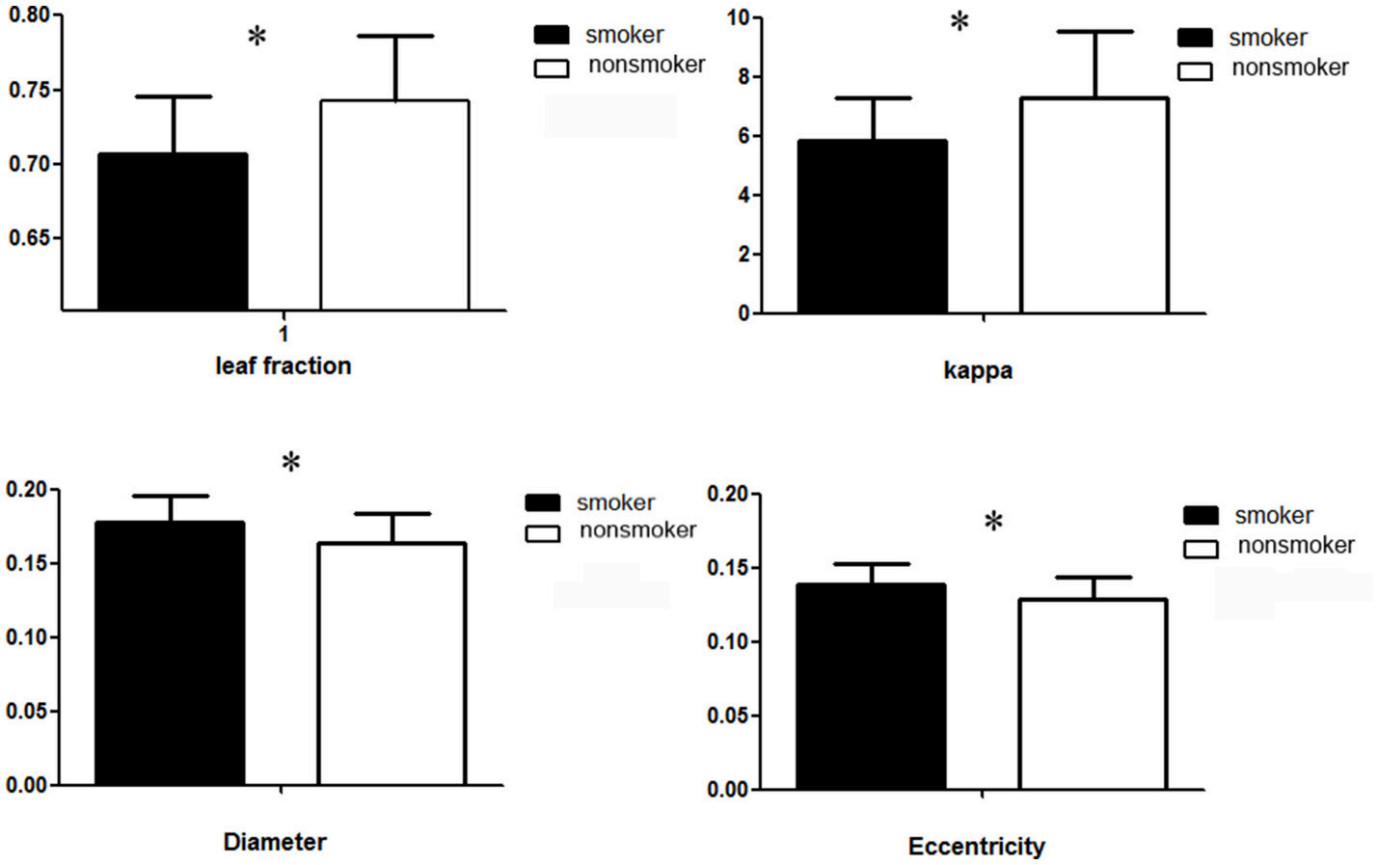
Group averages for leaf fraction, eccentricity, diameter and kappa measures of the MST. Open bars refer to nonsmoker and filled bars to young smoker (^*^p < 0.05).

Compared with nonsmokers, young smokers demonstrated decreased leaf fraction, kappa value, degree value and increased diameter, eccentricity value in alpha band (Table 2 and Figure 2). In detail, leaf fraction represented the whole tree distribution condition, which was decreased (*t* = −2.57, *p* = 0.009) in young smoker group. Diameter represented the small network short ways condition, which was increased (*t* = 2.387, *p* = 0.022) in young smoker. Eccentricity represented a tree leaf fraction and central correlation, which was increased (*t* = 2.371, *p* = 0.023) in young smoker. The kappa value responded to a tree high-degree and was decreased (*t* = −2.373, *p* = 0.023) in young smoker compare to nonsmoker. All changes between groups were shown in Figure 3. No significant changes in the delta, theta and beta band were found between the two groups.

**Table 2:** PLI average and MST measures.

		**Smoker**		**Control**		***T***	***P***
		***M***	***SD***	***M***	***SD***		
Delta	PLI	0.325	0.089	0.339	0.073	−0.147	0.595
	Degree	0.272	0.055	0.307	0.072	0.533	0.092
	Eccentricity	0.126	0.013	0.122	0.016	0.050	0.471
	BC	0.767	0.047	0.792	0.037	0.197	0.079
	Kappa	5.631	1.172	6.424	1.665	0.685	0.092
	Diameter	0.161	0.017	0.156	0.021	−0.255	0.439
	Leaf fraction	0.705	0.036	0.711	0.043	0.357	0.613
	Tree hierarchy	0.465	0.030	0.454	0.017	0.158	0.196
Theta	PLI	0.117	0.012	0.117	0.014	−0.147	0.884
	Degree	0.230	0.050	0.223	0.036	0.533	0.597
	Eccentricity	0.153	0.013	0.152	0.009	0.050	0.961
	BC	0.722	0.038	0.719	0.035	0.197	0.845
	Kappa	4.744	0.844	4.586	0.592	0.685	0.497
	Diameter	0.195	0.016	0.196	0.011	−0.255	0.800
	Leaf fraction	0.662	0.031	0.659	0.033	0.357	0.723
	Tree hierarchy	0.463	0.022	0.461	0.029	0.158	0.875
Alpha	PLI	0.189	0.094	0.218	0.096	−0.968	0.339
	Degree	0.280	0.074	0.344	0.097	−2.364	0.023
	**Eccentricity**	**0.139**	**0.013**	**0.128**	**0.015**	**2.371**	**0.023**
	BC	0.745	0.045	0.768	0.050	−1.530	0.134
	**Kappa**	**5.829**	**1.474**	**7.271**	**2.286**	−**2.371**	**0.023**
	**Diameter**	**0.178**	**0.018**	**0.164**	**0.020**	**2.387**	**0.022**
	**Leaf fraction**	**0.706**	**0.040**	**0.743**	**0.044**	−**2.759**	**0.009**
	Tree hierarchy	0.478	0.025	0.488	0.022	−1.419	0.164
Beta	PLI	0.078	0.013	0.081	0.014	−0.604	0.549
	Degree	0.227	0.029	0.258	0.065	−2.020	0.051
	Eccentricity	0.147	0.010	0.145	0.014	0.490	0.627
	BC	0.732	0.030	0.741	0.041	−0.786	0.437
	Kappa	4.690	0.563	5.342	1.353	−2.024	0.050
	Diameter	0.188	0.014	0.187	0.018	0.266	0.791
	Leaf fraction	0.671	0.028	0.680	0.036	−0.909	0.369
	Tree hierarchy	0.462	0.027	0.463	0.019	−0.099	0.922

*PLI, Phase lag index*.

*BC, Betweenness centrality*.

*Bold indicates significant difference, p < 0.05*.

### Correlation between brain and smoking behaviors

**Figure 4:**
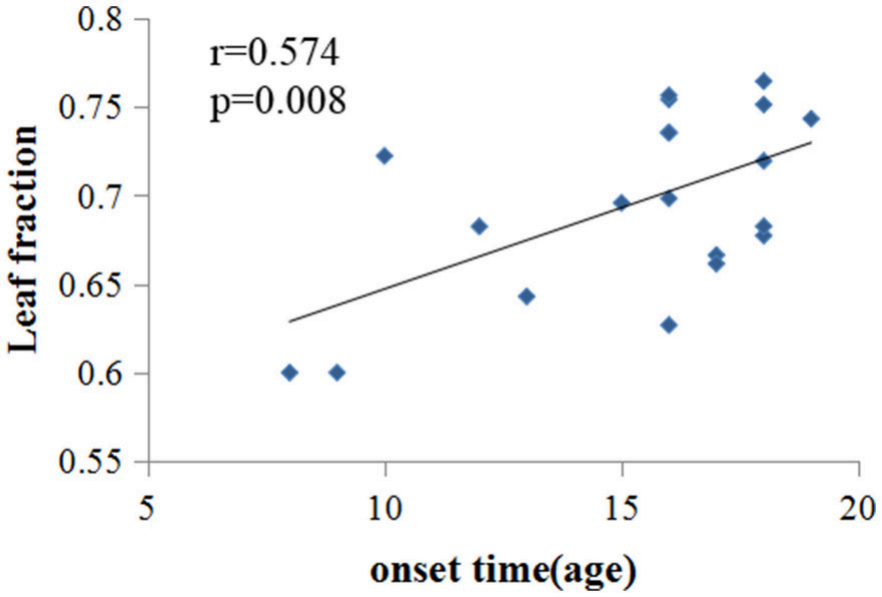
Significant correlation was found between leaf fraction and onset times in young smoker (*r* = 0.574, *p* = 0.008).

Pearson's correlation coefficients were calculated for alpha band (Eccentricity, Diameter, Leaf fraction, kappa) and smoking behaviors (pack-years, FTND, onset time (age), CPD). Significant correlation was found between leaf fraction and onset times (Figure 4, *r* = 0.574, *p* = 0.008) in smokers. No other significant correlations were found.

## Discussion

Complex brain networks may play a key role in the integrated processing of the brain function, which may be influenced by pathological processing. In present study, we analyzed EEG signals during the resting state to examine the topological characteristics of brain networks in young smokers by using PLI and MST methods. The major problem of EEG was volume conduction, which may cause overestimation of functional connectivity. The PLI measured the consistency of phase lags between EEG time series and provide more accurate results of functional connectivity (Stam et al., 2007; van Dellen et al., 2015; Yuan et al., 2016). Moreover, the MST method assessed the strongest connections in the network without forming loops, which solved the problem of arbitrary thresholding of connectivity values and preserved important information about network organization (Tewarie et al., 2015). Compared with nonsmokers, young smokers showed decreased leaf fraction, kappa value and increased diameter, eccentricity value in alpha band.

Leaf fraction indicates the extent of central organization in global network. High leaf fraction suggests that the communication of network is largely dependent on hub nodes. Kappa value indicates the broadness of the degree distribution (degree divergence). Diameter is the largest distance between any two nodes of the tree, which is related to the efficiency of global brain network organization. Information may be efficiently processed between remote brain regions with low diameter. In addition, eccentricity indicates longest distance between a reference node and any other node, which reflects the efficient information processing from the least central node (van Dellen et al., 2015; Fraga González et al., 2016). In present study, decreased leaf fraction, kappa value were found in young smoker, which were similar with previous EEG studies brain neural oscillations properties in smokers during the resting state (Rass et al., 2015). Decreased alpha band power in smokers might be associated with long-term consequences of nicotine use (Rass et al., 2015; Wilbanks et al., 2016). Despite of the significant advantages, the PLI analyses failed to reveal differences of the connectivity strength between nodes of the brain regions. The MST method avoided potential bias as thresholding value (Stam, 2014), which constructed of brain connectivity network (van Diessen et al., 2014). The MST analysis had been successfully applied to EEG data from several diseases (Delbeuck et al., 2003; van Dellen et al., 2014; Fraga González et al., 2016). In the current report, we found that smokers were associated with altered global brain networks. We also revealed the abnormal connectivity strength in the alpha band in young smokers by using the MST analyses. The optimal tree was configured by both short distances and no overload of the central tree nodes (Stam, 2014). In our study, the MST analysis presented us a disordered network of smokers as marked by decreases in leaf number indicating low network integration compare with nonsmokers. These results suggested a more path-like configuration in young smoker and a more star-like topology in nonsmoker in the tree topologies. The MST leaf fraction was lower for trees derived from regular networks and increased as these networks became more random and increased distances (Tewarie et al., 2015). Increased diameter value emerge to less communication between nodes of the network in smokers, which suggested the brain networks became more disorganized and decreased global efficiency in smokers. The eccentricity and kappa descript the configuration features of tree. In present study, increased eccentricity may reveal increased longest distance between a reference node and any other node in young male smokers, which reflects decreased efficient information processing from the least central node. Decreased kappa value was related to decreased broadness of the degree distribution, which may suggest slow synchronization of the tree in young male smokers (Stam and van Straaten, 2012).

Our results indicated less integrated network configuration in young smokers, which confirmed the decrease of global efficiency in alpha band in young smokers compared with nonsmokers. These findings may be associated with cholinergic deficits and cognitive impairment in young smokers. Positive correlation was found between leaf fraction and onset times in young smoker, which may suggest initial smoking was associated with the extent of central organization in global network. Young male smokers were associated with changes in brain network topology, including further decreased leaf number and increased eccentricity in the brain.

## Limitation

The study is limited in its cross-sectional design, and it is not possible to infer the causality of the relationships between alpha band and severity of smoking. The relationships might be bi-directional and related to some latent variables. In addition, our participants were all male. Previous studies revealed clear gender effect in smoking effects (Kühn et al., 2012; Franklin et al., 2014). Another limitation of the study is the relatively small sample size, so the results might not be generalize able to wider populations. Further longitudinal studies with larger sample size would be required to improve the understanding of the EEG findings in the current study.

## Conclusion

The abnormal topological characteristics of functional brain networks in alpha band were found in young smokers during the resting state. The findings may provide novel insights into the neural mechanism in smoking. We hoped our study could improve the understanding of smoking.

## Author contributions

SS, DY, KY, and XL conceived and designed the experiments; KY, SS, JC, YC, XZ, and YG performed the experiments; DY, KY, JC, YC, and TX analyzed the data; SS, DY, and KY wrote the article; YL and YB provided critical revision of the manuscript for important intellectual content. All authors critically reviewed content and approved final version for publication.

### Conflict of interest statement

The authors declare that the research was conducted in the absence of any commercial or financial relationships that could be construed as a potential conflict of interest.
